# Combined 2D-QSAR, Principal Component Analysis and Sensitivity Analysis Studies on Fluoroquinolones’ Genotoxicity

**DOI:** 10.3390/ijerph16214156

**Published:** 2019-10-28

**Authors:** Meijin Du, Dan Zhang, Yilin Hou, Xiaohui Zhao, Yu Li

**Affiliations:** 1 College of Environmental Science and Engineering, North China Electric Power University, Beijing 102206, China; mjdu0401@outlook.com (M.D.); hylshinanshen95@outlook.com (Y.H.); 1357joe@163.com (X.Z.); 2 MOE Key Laboratory of Resources and Environmental Systems Optimization, North China Electric Power University, Beijing 102206, China; 3 Jilin Province Shize Environmental Protection Technology Co., Ltd, Jilin 130012, China; zhangdan_1974@163.com

**Keywords:** fluoroquinolone molecules, genotoxicity, 2D-QSAR model, principal component analysis, sensitivity analysis, molecular design

## Abstract

In this paper, two-dimensional quantitative structure–activity relationship (2D-QSAR) and principal component analysis (PCA) methods were employed to screen the main parameters affecting the genotoxicity of fluoroquinolones (FQs), and the rules affecting the genetic toxicity of FQs were investigated by combining 2D-QSAR and PCA with the sensitivity analysis method. First, four types of parameters were calculated, namely, the geometric parameters (7), electronic parameters (5), physical and chemical parameters (8), and spectral parameters (7), but the physical and chemical parameters heat of formation (HF) and critical volume (CV) were excluded after the establishment of the 2D-QSAR model. Then, after PCA, it was found that the first principal component represented the main driving factors affecting the molecular genetic toxicity of FQs. In addition, after comprehensive analysis of the factor loading of the first, second, and third principal components, seven parameters affecting the genotoxicity of the FQs were screened out, namely, total energy (TE), critical temperature (CT), and molecular weight (Mol Wt) (increased with increasing genotoxicity of the FQs) and steric parameter (MR), quadrupole moment Q_XX_ (Q_XX_), quadrupole moment Q_YY_ (Q_YY_), and boiling point (BP) (decreased with increasing genotoxicity of the FQs); the above key parameters were also verified by sensitivity analysis. The obtained rules could be used to determine the substitution sites and the substitution groups associated with higher genotoxicity in the process of FQ modification, and these rules agreed well with the hologram quantitative structure–activity relationship (HQSAR) model. Finally, it was also found through SPSS analysis that the parameters screened in this paper were significantly correlated with FQ derivatives’ genetic toxicity.

## 1. Introduction

Fluoroquinolones (FQs) are derivatives of nalidixic acid or pyrazinoic acid and are synthetic bactericidal antibiotics with a common framework of a ketone acid [1]. FQs are widely used in human and veterinary antimicrobial drugs because of their broad-spectrum activity against gram-positive and gram-negative bacteria [2,3]. Genotoxicity refers to the toxic effects caused by physical and chemical factors in the environment that damage genetic material at the chromosomal, molecular, and nucleotide levels [4]. Aldred et al. [5] found that topoisomerase II (DNA gyrase) was the main target of quinolones in gram-negative bacteria and that topoisomerase IV was the inhibitory target of most quinolones in gram-positive bacteria. At present, the methods commonly used for detecting the genotoxicity of FQs are the Ames test [6], comet assay [7], micronucleus test [8], Ara test [9], and SOS/umu test (a short-term screening test for detecting environmental mutagenesis established and developed by Oda et al in 1985 based on the basic principle of umuC gene expression induced by SOS response during DNA damage) [10]. However, few studies on genotoxicity focus on the molecular structure of FQs themselves.

With the increasing abuse of antibiotics, a large number of these antibiotics enter the environment in the form of the original drugs or their metabolites [11,12]. In addition to causing chemical contamination, environmental pressures in the environment can increase the abundance of antibiotic resistance genes (ARGs), causing potential genetic pollution [13,14]. In contrast to traditional chemical pollutants, genetic pollutants are characterized by reproducibility, transmissibility, and environmental persistence and are more difficult to study and control [15]. Studies have shown that most FQ antibiotics remain in the environment and that residual antibiotics can be transported into the body through the food chain, adversely affecting the health of ecosystems [16], humans [17], and animals [18]. Therefore, it is particularly important to study the genotoxicity of FQ antibiotics and design new antibiotics to reduce the production of resistant genes [19]. The chemical structure of FQs not only determines their antibacterial activity but also is closely related to their adverse reactions [20]. Thus, it is important to understand their structure–activity relationships and structure–toxicity relationships.

The quantitative structure–activity relationship (QSAR) model uses theoretical calculations and statistical analysis tools to establish a quantitative relationship between the structural properties of a compound and its biological activity based on the correlation between the structure of a series of compounds with similar structural mechanisms and their biological activity [21]. Meijin et al. [22] used contour maps of a 3D-QSAR model to determine the molecular sites of hexachlorobenzene and designed 11 new low-migration hexachlorobenzene molecules. Yuanyuan et al. [23] determined nine single and double substitution sites based on the contour maps of electrostatic fields in the 3D-QSAR model and full factor experimental design and designed 34 new polychlorinated naphthalene molecules with low bioaccumulation. In addition, Xiaolei et al. [24] selected the R6 substituents of pentachlorophenol molecules according to the contour maps of the comparative molecular field analysis model (CoMSIA) which is a molecular field analysis method in the 3D-QSAR and designed seven new pentachlorophenol molecules with low biological enrichment. It can be seen that the use of contour maps in the 3D-QSAR model to identify molecular sites has become an important way to modify molecules. The contour maps of the 3D-QSAR model determine the molecular modification sites based on only the molecular structure and do not consider the effects of internal structure and external environment on the molecular properties. Therefore, finding a new molecular modification method to design environmentally friendly molecules is of great significance.

In this paper, a 2D-QSAR model was used to find the relevant parameters affecting the genotoxicity of FQs. Then, by principal component analysis (PCA), the main driving factors affecting the genotoxicity of FQs were identified, and the main influencing factors were found by combining the factor loading of each parameter. The sensitivity analysis method was used to analyze the sensitivity of all parameters and compare with the results of PCA to determine the main influencing parameters. By combining the positive and negative effects of various parameters in the 2D-QSAR model on the genotoxicity of FQs, the basic laws affecting the molecular genetic toxicity of FQs were determined, and verification was carried out from the perspective of substitution sites and substituent groups of FQs. This work provided theoretical support for the future study of FQ resistance genes and provided a new approach for molecular design, broadening the theoretical basis for the design of novel environmentally friendly FQs.

## 2. Materials and Methods

### 2.1. Data Sources and Calculation Methods

The genotoxicity data of FQ compounds towards *Salmonella typhimurium (*gram-negative bacteria) were obtained from the study by Min et al. [25] and expressed by pLOEC (-logLOEC). The geometric parameters, electronic parameters, physical and chemical parameters, and spectral parameters of FQs (Table S1) were calculated by Gaussian 2009 software (Gaussian corporation, USA) and Chemdraw12.0 software (CambridgeSoft corporation, USA) to study the genotoxic of FQs. The structures of 29 FQs are shown in the Table S2.

In this paper, we choose the Hartree–Fock method in the ab initio calculations, the Amifloxacin (AMI) method in the semi-empirical algorithm, and the density functional theory (selecting the B3LYP functional which is a very common functional in DFT theory) in the post self-consistent field (post-SCF) method to perform molecular structure optimization calculations at the 6–31G(d), 6–311++G(df, pd), 6–311+G(d, p), and 3–21G unit levels, respectively. On the basis of optimizing the structure, the bond lengths, bond angles, and dihedral angles of the Ofloxacin (OFL) molecule were calculated and compared with the experimental values. Through correlation analysis, it was found that the correlation coefficient R values between the experimental values and the calculated values from density functional theory DFT/B3LPY at the 6–31G(d), 6–311++G (df, pd), 6–311+G (d, p), and 3–21G unit levels were 0.99850, 0.99665, 0.99539, and 0.99381 and that those between the experimental values and the calculated values from the heat of formation (HF) were 0.99754, 0.99382, 0.99105, and 0.99558, respectively; the correlation coefficient R value for AMI was 0.99848. Among these methods, the DFT method at the B3LYP/6–31G(d) unit level provided the highest correlation coefficient R value. To obtain better accuracy, the B3LYP/6–31G(d) DFT method was used for the subsequent ab initio computations. The Molecular Mechanices 2 (MM2) method based on molecular mechanics embedded in the Chemdraw12.0 software (CambridgeSoft corporation, USA) system was used for dynamic simulation of molecular conformation, and the embedded GAMESS quantum chemical software (maintained by the members of the Gordon research group at Iowa State University) package was used to optimize the molecular conformation.

### 2.2. D-QSAR Model Analysis Method

The 2D-QSAR model analysis method uses the physicochemical parameters or structural parameter variables related to the compound to study the relationship between chemical substances and biological activity/toxicity by mathematical statistics; this method explains the changes in the physical and chemical properties of the compound caused by structural changes and how the biological activity of the compound is altered by these changes [26,27]. In this study, the genetic toxicity data (pLOEC) of FQs were selected as the dependent variable, and the geometric parameters, electronic parameters, physical and chemical parameters, and spectral parameters of FQs calculated using Gaussian 2009 software and Chemdraw12.0 software were used as the independent variables. A 2D-QSAR model between the genetic toxicity of FQs and their parameters was established by a multiple linear regression method to study the various parameters affecting the genotoxicity of FQs.

### 2.3. PCA Method

PCA is a statistical method that converts multiple indexes into a few comprehensive indexes and maintains a large amount of information from the original indexes [28]. PCA is widely used in the comprehensive evaluation of multiple indicators (variables) to eliminate the correlation between evaluation indicators and help find the main influencing factors more objectively [29]. In this paper, PCA was used to analyze relevant molecular parameters screened by the 2D-QSAR model and to find a few common factors controlling all variables to study the relationship between the molecular toxicity of FQs and various parameters.

### 2.4. Sensitivity Analysis

Sensitivity analysis is a method of studying and analyzing the sensitivity of a system (or model) state or output change to changes in system parameters or surrounding conditions [30]. The main parameters, which were initially screened by the 2D-QSAR model and finalized by PCA, could be verified by the calculation of the sensitivity parameters to more accurately analyze the influence of the main parameters on the genotoxicity of FQs. The sensitivity coefficient is defined as the ratio of the relative change in the predicted value to the relative change in the input parameter, i.e.,

(1) $${SC}_{i}=\left(\Delta Y_{i}/Y_{i}\right)/\left(\Delta X_{i}/X_{i}\right)$$

In Equation (1), SC_i_ represents the sensitivity coefficient of the input parameter, and ΔX_i_/X_i_ and ΔY_i_/Y_i_ represent the rates of change in the input parameter and the corresponding prediction result, respectively.

## 3. Results and Discussion

### 3.1. Molecular Genotoxicity Analysis of FQs Based on the 2D-QSAR Model

To construct the 2D-QSAR model between the molecular toxicity of the FQs and their parameters, the genotoxicity data of the FQs were used as the dependent variable, and the FQ parameters were used as the independent variable. Multiple linear regression was used to study various parameters affecting the molecular genetic toxicity of FQs. The spectral parameters of FQs mainly included positive frequency (PF), IR C–O stretching vibration frequency (IR-(C–O)svf), IR benzene ring breathing vibration frequency (IR-brbvf), IR molecular breathing vibration frequency (IR-mbvf), Raman C–O stretching vibration frequency (Raman-(C–O)svf), Raman benzene ring breathing vibration frequency (Raman-brsvf), and Raman molecular breathing vibration frequency (Raman-msvf). The geometric parameters of FQs mainly included quadrupole moment Q_XX_ (Q_XX_), quadrupole moment Q_YY_ (Q_YY_), quadrupole moment Q_ZZ_ (Q_ZZ_), quadrupole moment Q_XY_ (Q_XY_), quadrupole moment Q_YZ_ (Q_YZ_), steric parameter (MR), and molecular weight (Mol Wt). The electronic parameters of FQs mainly included total energy (TE), the most positive atomic partial Mulliken charge (q^+^), the most negative atomic partial Mulliken charge (q^−^), the lowest unoccupied molecular orbital energy (*E*_LUMO_), and energy gap (EG). The physical and chemical parameters of FQs mainly included boiling point (BP), melting point (MP), critical temp (CT), critical vol (CV), oil–water distribution coefficient (log*P*), Henry’s law constant (HL), heat of formation (HF), and Gibbs energy (GE). The calculated values of each parameter of FQs are shown in Table 1. The equation for the relationship between the genetic toxicity and parameters of FQs could be deduced as follows:

(2) $$\begin{array}{l} \mathrm{pLOEC}=-223.144+0.021\mathrm{TE}-50.087q^{+}-12.054q^{-}-521.252E_{\mathrm{LUMO}}+625.180\mathrm{EG}-0.085\mathrm{PF}\\ -0.022Q_{\mathrm{XX}}+0.098Q_{\mathrm{YY}}+0.001Q_{\mathrm{ZZ}}-0.048Q_{\mathrm{XY}}+0.639Q_{\mathrm{YZ}}-0.112\mathrm{BP}-0.017\mathrm{MP}\\ +0.356\mathrm{CT}+0.014\mathrm{GE}+1.052\mathrm{logP}-1.639\mathrm{MR}+4.406\mathrm{HL}+0.403\mathrm{MolWt}-0.012\mathrm{IR}\text{-}(C\text{-}O)\mathrm{svf}\\ -0.007\mathrm{IR}\text{-}\mathrm{brbvf}-0.005\mathrm{IR}\text{-}\mathrm{mbvf}-1.501\mathrm{Raman}\text{-}(C\text{-}O)\mathrm{svf}-0.375\mathrm{Raman}\text{-}\mathrm{brbvf}-0.289\mathrm{Raman}\text{-}\mathrm{mbvf} \end{array}$$

In Equation (2), the correlation coefficient R was 1.000 (>0.8), and Sig was 0.00 (<0.05), passing the significance test [31], which indicated that all parameters in the 2D-QSAR model were related to the molecular toxicity of FQs. The correlation coefficient q^2^ of the model was 0.78, and the ratio of R^2^ and q^2^ was 22% (<25%) indicating that the model was not overfitted [32]. Among all the parameters, HF and CV were excluded by regression analysis, showing that the effect of these two parameters on the genetic toxicity of FQ molecules could be neglected. By analyzing the equation between the genetic toxicity and the parameters of FQs, it can be seen that the coefficients before TE, EG, Q_YY_, Q_ZZ_, Q_XZ_, Q_YZ_, CT, GE, log*P*, HL, and Mol Wt were all positive, indicating that they had positive effects on the genetic toxicity of FQs; that is, the genotoxicity of FQs showed an increasing trend with increasing TE, EG, Q_YY_, Q_ZZ_, Q_XZ_, Q_YZ_, CT, GE, log*P*, HL, or Mol Wt. In addition, the coefficients before q^+^, q^−^, *E*_LUMO_, PF, Q_XX_, Q_XY_, BP, MP, MR, IR-(C–O) svf, IR-brbvf, IR-mbvf, Raman-(C–O) svf, Raman-brsvf, and Raman-msvf were all negative, indicating that they had negative effects on the genetic toxicity of FQs (i.e., with increasing q^+^, q^−^, *E*_LUMO_, PF, Q_XX_, Q_XY_, BP, MP, MR, IR-(C–O) svf, IR-brbvf, IR-mbvf, Raman-(C–O) svf, Raman-brsvf, or Raman-msvf, the genotoxicity of FQs showed a decreasing trend).

### 3.2. Analysis of FQ Genotoxicity Based on Principal Component Analysis

PCA was carried out for the parameters (TE, q^+^, q^−^, *E*_LUMO_, EG, PF, Q_XX_, Q_YY_, Q_ZZ_, Q_XY_, Q_YZ_, BP, MP, CT, GE, log*P*, MR, HL, Mol Wt, IR-(C–O) svf, IR-brbvf, IR-mbvf, Raman-(C–O) svf, Raman-brsvf, and Raman-msvf) screened by 2D-QSAR that were correlated with the genetic toxicity of FQs. The Kaiser–Meyer–Olkin (KMO) test result for the partial correlation between test variables was found to be 0.798 (>0.7), indicating that the correlation between variables was relatively high and that the data were suitable for PCA [33]. The results of the spherical test showed that the significance level of the observation was 0.00 < 0.05 [34] under the null hypothesis that the correlation coefficient matrix was an identity matrix. Therefore, the null hypothesis that all variables were independent of each other was rejected, indicating that at least two of these variables were related; that is, there was a simple linear correlation between them, which could be used for PCA.

**Figure 1:**
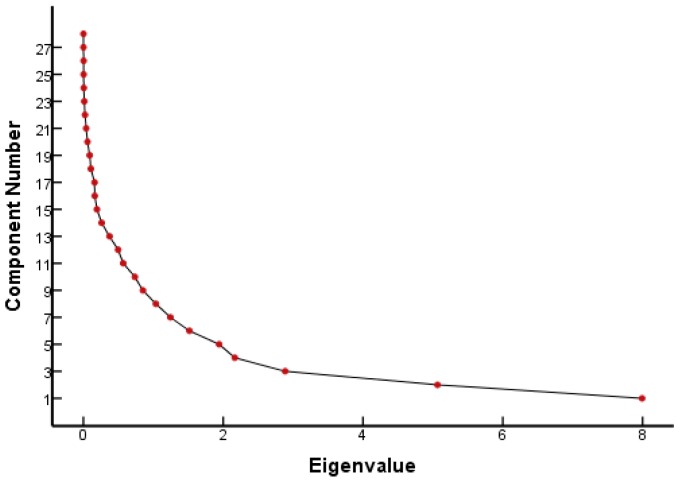
Scree plot of FQ parameters.

From the FQ parameter scree plot (Figure 1), it can be seen that there were eight principal components whose eigenvalues were greater than 1 and that the inflection point of the polyline started from the eighth principal component. Combined with the eigenvalues and interpretation (Table 1), eight principal components were selected to analyze the parameters that played a major role in the genetic toxicity of FQs, which could explain 86.18% of the original variable information. Table 1 lists the results of the PCA for each parameter of the FQs.

Factor loading analysis (Table 1) showed that TE, Q_XX_, Q_YY_, BP, CT, MR, and Mol Wt had higher loadings in the first principal component and that their absolute values were 0.828, 0.888, 0.885, 0.853, 0.827, 0.947, and 0.97, respectively, all of which were greater than 0.8. These parameters mainly reflected the internal structure and critical temperature of the molecule, which could be called the molecular structure and critical temperature factor of FQs. Moreover, the eigenvalue of the first principal component was 7.992, with a contribution rate of 28.54%, which was higher than that of the other principal components, indicating that the structure of FQs had a greater impact on the molecules themselves than did the other parameters. The contribution rate of the second principal component was 18.09%, and the eigenvalue was 5.066. The absolute values of the loading of IR-(C–O) svf and Raman-(C–O) svf were 0.796 and 0.778, respectively, both of which were higher than 0.7. IR-(C–O) svf and Raman-(C–O) svf mainly represented spectral information of molecules, so the second principal component could be called the spectral factor. In the third principal component, the principal component contribution rate was 10.31%, and the eigenvalue was 2.887. The absolute value of factor loading of GE, HL and IR-mbvf was relatively high, 0.735, 0.659 and 0.608, respectively, all of which were greater than 0.6. These parameters mainly represent the internal energy of molecules, indicating that the third principal component was the internal energy factor of molecules. In the fourth principal component, the contribution rate and eigenvalue were 7.74% and 2.166, respectively. The absolute values of factor loading of q^+^, q^−^, and IR-brbvf were relatively high—0.543, 0.693, and 0.602, respectively—all of which were greater than 0.5, which mainly reflected the electronic information about FQs. Therefore, the fourth principal component corresponds to the molecular electronic structure factor. However, the principal component loadings of the fifth, sixth, seventh, and eighth principal components were 6.93%, 5.42%, 4.44%, and 3.707, respectively, which were all lower than the first to the fourth principal component, showing a relatively small impact on the molecular genetic toxicity of FQs.

Through PCA of all parameters, it was found that the factor contribution rates of the first, second, and third principal components were relatively high, reaching 56.95% accumulatively, among which the interpretation degrees of the molecular structure and critical temperature factor (28.54%) were the largest, indicating these parameters as the main driving factors affecting the molecular genetic toxicity of FQs. In this paper, the parameters TE (−0.828), Q_XX_ (−0.888), Q_YY_ (−0.885), BP (0.853), CT (0.827), MR (0.947), and Mol Wt (0.97) in the first principal component; IR-(C–O) svf (0.796) and Raman-(C–O) svf (0.778) in the second principal component; and GE (0.735), HL (0.659), and IR-mbvf (0.608) in the third principal component were selected as the main parameter factors. Through a comprehensive analysis of parameter factor loading, it was found that the absolute factor loading values of the parameters TE, Q_XX_, Q_YY_, BP, CT, MR, and Mol Wt were all greater than 0.8 and were all in the first principal component, indicating that these seven parameters had a large proportion of influence on the genetic toxicity of FQs and were important parameters to influence the genetic toxicity of FQs.

### 3.3. Genotoxicity Parameter Verification of FQs Based on Sensitivity Analysis

PCA found that TE, Q_XX_, Q_YY_, BP, CT, MR, and Mol Wt played an important role in the genotoxicity of FQs. To further explain the influence of the above parameters on the genotoxicity of FQs, sensitivity analyses were performed on all parameters in the equation by combining the equation between the toxicity of FQs and their parameters derived from the 2D-QSAR model. The sensitivity coefficient values of each parameter for the genotoxicity of FQs were calculated, and each parameter was increased by 10%, 20%, 30%, 40%, and 50%. The degree of influence on the genotoxicity was calculated by the change in parameters, and the influence trend and significance of each parameter on the results were expressed by the relative sensitivity. The sensitivity coefficients of each parameter are shown in Table 2.

**Figure 2:**
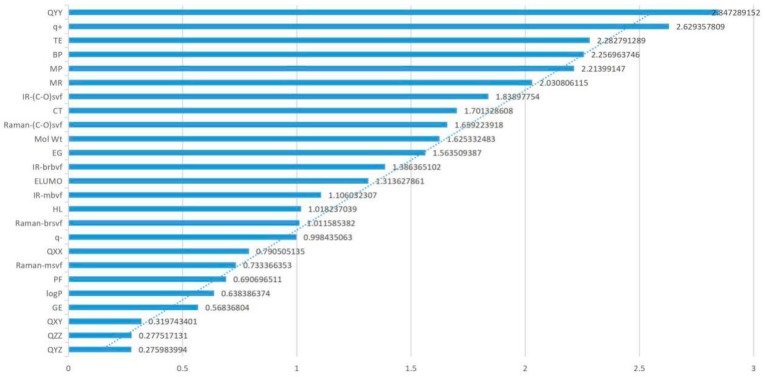
Genotoxicity sensitivity coefficient of FQ parameters.

Through the sensitivity analysis of all parameters, the degree of influence on the genotoxicity of FQs was calculated under the condition of 10%, 20%, 30%, 40%, and 50% changes in each parameter. To more directly study the influence degree of each parameter on genetic toxicity, the absolute value of the average sensitivity coefficient under different change degrees of each parameter was taken for analysis. Since the absolute value of the average sensitivity coefficient value of each parameter differs greatly, this paper took the 1/3 power of the absolute value of the average sensitivity coefficient value of the parameter for analysis (Figure 2). The effect of each parameter on the genotoxicity of FQs can be clearly seen in Figure 2. Among the parameters TE, Q_XX_, Q_YY_, BP, CT, MR, and Mol Wt, the absolute values of the Q_YY_, TE, BP, and MR sensitivity coefficients were greater than 0.2, and the absolute value of the Q_YY_ sensitivity coefficient was the highest. The CT and Mol Wt sensitivity coefficient absolute values were greater than 0.15 and compared to the absolute value of the sensitivity coefficients of the remaining six parameters, the Q_XX_ sensitivity coefficient absolute value was relatively low but close to 1. This finding indicated that the main parameters screened by PCA also play an important role in the 2D-QSAR model and that the influence of the seven parameters on the genetic toxicity of FQs cannot be ignored.

### 3.4. FQ Genotoxicity and Mechanism Analysis

#### 3.4.1. The Change Law of Molecular Genotoxicity of FQs

The main parameters influencing the molecular genotoxicity of FQs were screened by PCA, including TE, Q_XX_, Q_YY_, BP, CT, MR, and Mol Wt. Combined with the positive and negative effects of 2D-QSAR model independent variables on the genotoxicity of FQs, it was found that the change law influencing the molecular genetic toxicity of FQs was as follows: the genotoxicity of FQs decreased with increasing MR, Q_XX_, Q_YY_, and BP, but with increasing TE, CT, and Mol Wt, the genotoxicity of FQs increased.

Xiaohui et al. [35] used the hologram quantitative structure–activity relationship (HQSAR) method to construct a quantitative model of the structure–activity relationship between the genotoxicity of quinolones towards gram-negative bacteria and the structure of these quinolones. Amifloxacin (AMI), balofloxacin (BAL), cinoxacin (CIN), fleroxacin (FLE), pazufloxacin (PAZ), and rufloxacin (RUF) were selected as target molecules, and their C-7 position was modified to design 35 novel quinolones with improved genotoxicity against gram-negative bacteria. In the present paper, the target molecule selected by Xiaohui et al. [35] was taken as an example to determine the modification sites and select substituents of the target molecule based on the law affecting the genetic toxicity of FQs to verify the reliability of the above law.

(1) Consistency with FQ genotoxic HQSAR model modification sites.

**Figure 3:**
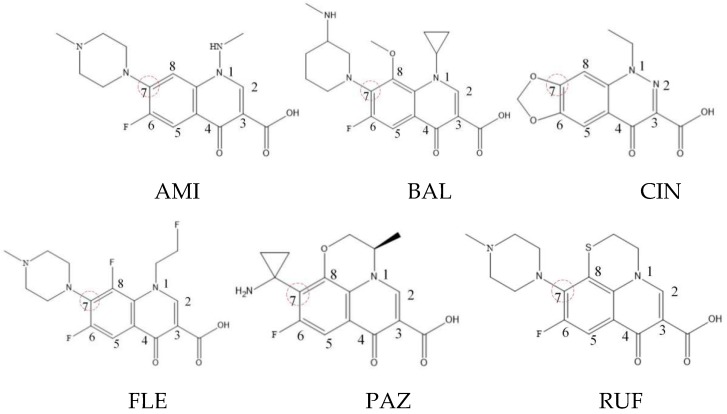
Amifloxacin (AMI), balofloxacin (BAL), cinoxacin (CIN), fleroxacin (FLE), pazufloxacin (PAZ), and rufloxacin (RUF) molecular structures and modified substitution sites.

The steric effect of the molecule was closely related to its molecular volume. As the volume of the group increased, the molecular stereoscopic effect also increased [36]. According to the molecular structure diagrams of AMI, BAL, CIN, FLE, PAZ, and RUF (Figure 3), the group volume connected by site 7 of FQs was the largest among the modification sites. Therefore, if the molecular stereoscopic effect was reduced, that is, the genotoxicity of the molecule was increased, the preferred substituted group was the group to which the seventh position was attached. This assessment was consistent with the rule of the substitution site (No. 7) of FQ molecular genotoxicity determined by the activity contribution diagram from the HQSAR model [34].

The quadrupole moment represents the asymmetric distribution of molecular charge on a three-dimensional sphere [37], and Q_XX_ and Q_YY_ represent the contribution of the charge system to the quadrupole moment in the x and y principal directions, respectively. By analyzing the electronic structure of FQs and integrating the charge distribution along the x and y principal axes, it was found that site 7 was the key site affecting the polar moment of molecules Q_XX_ and Q_YY_. Therefore, to reduce molecular Q_XX_ and Q_YY_ (to improve molecular genetic toxicity), the group which preferred substitution was the group connected at point 7. This result was consistent with the rule of the substitution site (No. 7) of FQs with high genotoxicity determined by Xiaohui et al. [35] through the activity contribution diagram from the HQSAR model.

(2) Consistency with the influence of FQ substituent groups on genotoxicity.

**Figure 4:**
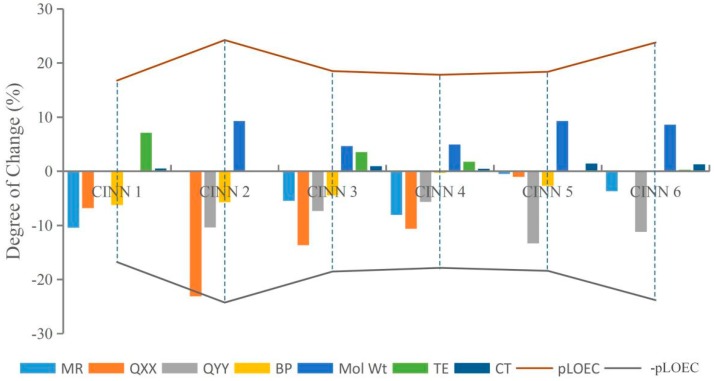
The variation trend of CIN molecular derivative parameters and their genotoxicity.

This paper randomly selected CIN molecular derivatives (introducing those with 1-methylpiperazine, 1-ethyl-4-methylpiperazine, 1,3-dimethylpiperazine, 1-methylpiperidine-4-alcohol, 1,3,5-trimethylpiperazine, and 1-methyl-3-cyclopropyl-4-aminopiperidine at position 7 of the CIN molecule) designed by Xiaohui [35] for analysis. The parameters TE, Q_XX_, Q_YY_, BP, CT, MR, and Mol Wt of each novel CIN molecule were calculated, as shown in Table 3. To further investigate whether the effects of different substituent groups on molecular genotoxicity were consistent with the rules found in this paper, the maximum or minimum parameter values of various parameters of the novel CIN molecule were used as benchmarks, and the degree of decrease or increase in each parameter to its maximum or minimum value was calculated and compared with its genotoxicity value, as shown in Figure 4. It was found that the genotoxicity trend of new CIN molecules was consistent with the increasing or decreasing trend of each parameter; that is, the greater the decline in the parameters TE, Q_XX_, Q_YY_, and BP of each molecule, the greater the increase in the parameters CT, MR, and Mol Wt, and the more obvious the increase in the toxicity value of the molecule, which was consistent with the law of genetic toxicity of FQs found in this paper. The basic rules of molecular modification found in this paper involve screening a large number of parameters by the 2D-QSAR model and PCA and verifying by sensitivity analysis, which is feasible to provide important theoretical support for the design of environmentally friendly molecules in different settings in the future and to some extent broaden the theoretical basis for the design of new environmentally friendly FQs.

#### 3.4.2. Correlation Analysis between the Main Parameters and Genotoxicity of FQ Derivatives and Their Tautomeric Forms

In this paper, the variation trend of CIN molecular derivative parameters and their genotoxicity was analyzed, and it was found that the trend of genotoxicity of new CIN molecules was consistent with the trend of increasing or decreasing the degree of each parameter. To further study the influence of the screened parameters on the genetic toxicity of FQ derivatives, SPSS software was used in this paper to analyze the correlation between the TE, Q_XX_, Q_YY_, BP, CT, MR, and Mol Wt parameter values of each CIN molecular derivative and their genotoxicity values. The TE, Q_XX_, Q_YY_, BP, CT, MR, and Mol Wt parameter values and genetic toxicity values of CIN molecular derivatives are shown in Table 3. It was found that the correlation coefficients R^2^ were all 1 (>0.9, close to 1.00) and that Sig was 0.00 (<0.05), indicating that the screened parameters were highly correlated with the genetic toxicity of FQ derivatives.

The tautomeric forms of quinolones were randomly selected for model verification. The parameters (TE, Q_XX_, Q_YY_, BP, CT, MR, and Mol Wt) of five tautomeric forms were calculated (Table 4). On the one hand, the 2D-QSAR model of FQ genotoxicity was constructed according to the seven parameters screened in this paper to calculate the genotoxicity values of tautomeric forms. On the other hand, the genotoxicity values of tautomeric forms were predicted by the HQSAR model [35]. Comparing the genotoxicity values from 2D-QSAR with the genotoxicity values from HQSAR reveals that the degrees of change were all below 10%, indicating that the main parameters obtained in the final screening of this paper also had good explanatory ability for the tautomeric forms of FQs.

## 4. Conclusions

The geometric parameters, physical and chemical parameters, electronic parameters, and spectral parameters of FQs were calculated systematically, and all parameters affecting the genetic toxicity of FQs were analyzed by the 2D-QSAR model, PCA, and sensitivity analysis. It was found that the structure and critical temperature parameters were the main driving factors affecting the genetic toxicity of FQs and that the parameters TE, Q_XX_, Q_YY_, BP, CT, MR, and Mol Wt were the main influencing parameters. Moreover, the genotoxicity of FQs decreased with increasing MR, Q_XX_, Q_YY_, and BP but increased with increasing TE, CT, and Mol Wt, which was verified by the relevance of substitution sites and substituent groups and the relationship between the parameters and genotoxicity. The genetic toxicity rules of FQs found in this paper can provide theoretical support for future research on the genetic toxicity of FQs and to some extent broaden the theoretical basis of the molecular design of new environmentally friendly FQs.

## Figures and Tables

**Table 1:** Geometric parameter, electronic parameter, physical and chemical parameter, and spectral parameter factor loading of fluoroquinolones (FQs).

Parameters	F1	F2	F3	F4	F5	F6	F7	F8
TE (aU)	−0.828	0.208	0.118	0.113	0.012	0.266	0.003	0.155
q+ (e)	−0.324	−0.617	0.196	0.543	−0.07	0.145	−0.249	−0.178
q^−^ (e)	−0.328	0.43	−0.127	0.693	0.17	0.079	−0.057	0.111
*E*LUMO (eV)	0.086	0.633	0.205	−0.072	0.095	0.563	0.063	0.375
EG (eV)	−0.068	0.626	0.139	0.024	−0.221	0.229	0.218	−0.461
PF (cm^−^^1^)	0.215	0.26	0.49	−0.283	0.073	0.526	0.113	−0.144
Q_XX_ (Debye·Å)	−0.888	−0.032	−0.084	0.137	−0.184	−0.03	0.183	0.013
Q_YY_ (Debye·Å)	−0.885	−0.181	0.027	−0.233	−0.169	0.143	−0.085	0.032
Q_ZZ_ (Debye·Å)	−0.174	−0.057	0.225	0.122	0.499	−0.267	0.657	0.073
Q_XY_ (Debye·Å)	0.116	−0.432	−0.169	−0.205	0.572	0.136	−0.469	−0.015
Q_YZ_ (Debye·Å)	−0.003	−0.467	−0.005	−0.324	0.688	−0.043	−0.022	0.164
BP (K)	0.853	−0.288	0.265	0.039	−0.062	0.01	−0.02	−0.148
MP (K)	0.585	−0.465	0.487	0.037	−0.253	0.039	0.004	−0.076
CT (K)	0.827	−0.221	0.382	0.147	0.008	−0.039	−0.094	0.069
GE (kJ/mol)	−0.224	0.036	0.735	0.124	0.105	−0.17	−0.275	0.283
logP	0.594	−0.051	−0.154	0.288	0.385	0.099	0.224	−0.08
MR (cm^3^/mol)	0.947	0.161	0.162	0.077	0.089	0.089	−0.071	0.055
HL	−0.372	−0.403	0.659	0.317	0.085	−0.054	−0.112	−0.223
Mol Wt	0.97	0.039	−0.044	0.046	−0.032	0.003	0.038	−0.048
IR-(C–O)svf (cm^−^^1^)	−0.178	0.796	0.352	−0.053	0.231	−0.229	−0.078	−0.249
IR-brbvf (cm^−^^1^)	−0.22	−0.404	0.049	0.602	0.187	0.452	0.09	−0.073
IR-mbvf (cm^−^^1^)	−0.039	−0.498	0.608	−0.135	−0.087	−0.194	0.414	0.136
Raman-(C–O)svf (cm^−^^1^)	−0.068	0.778	0.362	0.045	0.075	−0.401	−0.172	−0.013
Raman-brsvf (cm^−1^)	0.478	0.497	−0.05	0.278	−0.188	−0.24	−0.167	0.003
Raman-msvf (cm^−1^)	0.432	0.066	−0.07	0.426	−0.341	−0.049	0.056	0.521
Eigenvalue	7.992	5.066	2.887	2.166	1.941	1.516	1.244	1.038
Contribution rate %	28.54	18.09	10.31	7.74	6.93	5.42	4.44	3.71
Cumulative contribution rate %	28.54	46.64	56.95	64.68	71.62	77.03	81.48	85.18

Note: TE: total energy; q^+^: the most positive atomic partial Mulliken charge; q^−^: the most negative atomic partial Mulliken charge; E_LUMO_: the lowest unoccupied molecular orbital energy; EG: energy gap; PF: positive frequency; Q_XX_: quadrupole moment Q_XX_; Q_YY_: quadrupole moment Q_YY_; Q_ZZ_: quadrupole moment Q_ZZ_; Q_XY_: quadrupole moment Q_XY_: Q_YZ_: quadrupole moment Q_YZ_; BP: boiling point; MP: melting point; HL: Henry’s law constant; Mol Wt: molecular weight; IR-(C–O)svf: IR C–O stretching vibration frequency; IR-brbvf: IR benzene ring breathing vibration frequency; IR-mbvf: IR molecular breathing vibration frequency; Raman-(C–O)svf: Raman C–O stretching vibration frequency; Raman-brsvf: Raman benzene ring breathing vibration frequency; Raman-msvf: Raman molecular breathing vibration frequency.

**Table 2:** Calculation of the sensitivity coefficient of the independent variables (parameters) in the two-dimensional quantitative structure–activity relationship (2D-QSAR) model.

Parameter	10%	20%	30%	40%	50%
TE (aU)	−6.1400	−15.1579	62.3985	11.5861	6.7924
q^+^ (e)	−5.7542	−15.2817	93.7658	11.2650	6.8956
q^−^ (e)	0.9945	0.9950	0.9954	0.9957	0.9960
*E*_LUMO_ (eV)	2.7980	2.4334	2.1917	2.0198	1.8912
EG (eV)	5.5865	4.5002	3.3751	3.0462	2.6025
PF (cm^−^^1^)	−0.2641	−0.2951	−0.3278	−0.3622	−0.3984
Q_XX_ (Debye·Å)	0.4538	0.4755	0.4955	0.5140	0.5312
Q_YY_ (Debye·Å)	−2.6409	−3.7918	−6.0044	−12.0210	−90.9575
Q_ZZ_ (Debye·Å)	−0.0180	−0.0197	−0.0214	−0.0230	−0.0247
Q_XY_ (Debye·Å)	−0.0275	−0.0301	−0.0327	−0.0353	−0.0379
Q_YZ_ (Debye·Å)	−0.0176	−0.0193	−0.0210	−0.0227	−0.0245
BP (K)	35.0316	9.1324	5.6180	4.2245	3.4770
MP (K)	−2.4720	−3.4785	−5.3066	−9.6565	−33.3486
CT (K)	9.0000	5.4000	4.0345	3.3158	2.8723
GE (kJ/mol)	−0.1507	−0.1667	−0.1831	−0.2000	−0.2174
log*P*	0.2299	0.2456	0.2608	0.2753	0.2893
MR (cm^3^/mol)	21.4571	7.9357	5.1740	3.9862	3.3241
HL	1.0656	1.0598	1.0550	1.0508	1.0473
Mol Wt	7.2162	4.7536	3.6888	3.0944	2.7153
IR-(C–O)svf (cm^−^^1^)	−4.0549	−7.0062	−18.2386	48.7423	11.6530
IR-brbvf (cm^−^^1^)	−1.4838	−1.8711	−2.4015	−3.1722	−4.3945
IR-mbvf (cm^−^^1^)	−0.9196	−1.0947	−1.3049	−1.5621	−1.8839
Raman-(C–O)svf (cm^−^^1^)	−3.2035	−4.9392	−9.0428	−32.1439	26.4899
Raman-brsvf (cm^−^^1^)	−0.7395	−0.8656	−1.0101	−1.1803	−1.3803
Raman-msvf (cm^−^^1^)	0.3564	0.3765	0.3956	0.4133	0.4302

**Table 3:** The structure and main parameters of CIN molecular derivatives.

Compound	Structure	MR (cm^−3^/mol)	Q_XX_ (Debye·Å)	Q_YY_ (Debye·Å)	BP (K)	Mol Wt	TE (aU)	CT (K)	pLOEC	ΔpLOEC
CINN 1	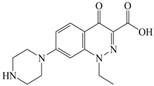	84.38	−158.28	−123.36	955.4	302.33	−1027.08	961.98	7.837	1.317
CINN 2	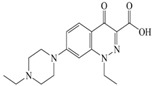	94.22	−182.41	−136.16	960.78	330.38	−1105.71	957.05	8.611	2.091
CINN 3	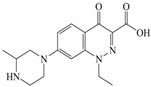	89.07	−168.43	−132.4	973.61	316.35	−1066.41	966.16	8.006	1.486
CINN 4	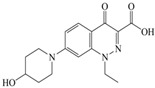	86.61	−163.89	−130.37	1017.24	317.34	−1086.28	961.41	7.939	1.419
CINN 5	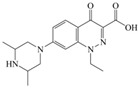	93.76	−149.72	−139.78	991.82	330.38	−1105.73	970.94	7.993	1.473
CINN 6	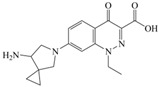	90.77	−148.17	−137.17	1018.91	328.37	−1104.48	969.46	8.56	2.04

**Table 4:** The parameters and genotoxicity values of five tautomeric forms.

Structures	Parameters							Genotoxicity Values		
	Mol Wt	BP (K)	CT (K)	MR	TE (aU)	Q_XX_ (Debye·Å)	Q_YY_ (Debye·Å)	2D-QSAR	HQSAR	Degree of Change (%)
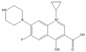	332.35	987.79	965.51	90.78	−1148.32	−17.0258	10.1843	4.0635	4.3154	5.84
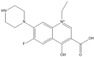	320.34	958.27	948.57	88.37	−1110.25	−17.9215	9.0487	4.2216	4.6281	8.78
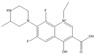	352.15	980.63	949.98	93.46	−1248.79	−15.0983	5.6736	4.9677	5.2607	5.57
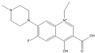	334.16	940.67	938.98	93.41	−1149.56	−19.7039	10.1977	4.7043	5.0496	6.84
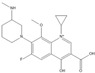	390.43	1080.78	999.32	107.12	−1341.44	−9.4622	5.3841	4.5417	4.9107	7.51

## Supplementary Materials

The following are available online at https://www.mdpi.com/1660-4601/16/21/4156/s1, Table S1: The spectral parameters, geometric parameters, electronic parameters, and physical and chemical parameters and the calculated values for FQs, Table S2: The structures of 29 FQs.
