# Supplementary material for: Combined 2D-QSAR, Principal Component Analysis and Sensitivity Analysis Studies on Fluoroquinolones’ Genotoxicity
Source: Int J Environ Res Public Health. 2019 Oct 28;16(21):4156. doi: 10.3390/ijerph16214156 (PMC6862474; doi:10.3390/ijerph16214156)
Supplement: Supplementary file 1 [file ijerph-16-04156-s001.pdf]

Supplementary materials

Table 1. The spectral parameters, geometric parameters, electronic parameters, and physical and chemical parameters and the calculated values for FQs.

Parameters\FQs		Dif	Enr	Nor	Lom	Ofl	Pef	Fle	Cip	Bal	Mar	Pip	Cin	Eno	Dan	Gat	Lev	Ruf	Paz	Nad	Mox	Spa	Sar	Ami	Bes	Cli	Gre	Orb	Sit	Tem
Spectral	PF (cm^{-1})	15.72	22.33	25.15	15.18	14.35	22.04	17.05	28.00	24.33	21.00	22.88	-14.01	27.27	24.98	25.42	25.98	25.68	31.13	24.39	18.73	21.17	15.95	24.59	14.17	29.96	24.90	26.90	13.46	22.20
	IR-(C–O)svf (cm^{-1})	1892.15	1797.61	1771.29	1775.59	1810.54	1792.12	1757.45	1788.85	1829.69	1916.33	1713.14	1413.04	1737.15	1788.15	1838.51	1806.36	1750.14	1747.40	1797.15	1824.60	1326.55	1888.46	1768.34	1767.64	1749.28	1718.58	1845.52	1256.36	861.26
	IR-brbvf (cm^{-1})	1536.73	1289.58	1180.48	1788.98	794.80	1587.11	937.03	1247.65	1366.78	606.27	2430.37	1631.51	1450.51	1512.57	1363.81	964.12	776.83	800.36	1623.97	849.79	1185.52	1539.67	1290.74	898.02	900.21	1488.51	1280.91	1141.39	2155.95
	IR-mbvf (cm^{-1})	870.07	1198.96	997.32	461.74	1428.91	909.28	444.43	1031.78	671.03	971.45	2049.69	826.61	3427.60	1450.26	1161.31	518.38	1015.14	949.78	684.26	1325.30	2115.20	812.93	1140.99	1682.72	1571.06	725.70	848.34	1694.43	1200.51
	Raman-(C–O)svf (cm^{-1})	13.90	12.00	11.25	11.70	13.32	12.38	12.00	11.57	12.42	14.45	10.96	9.05	11.02	12.47	12.23	13.82	11.75	10.13	11.06	17.71	8.30	13.58	12.56	11.19	11.30	11.90	11.77	6.29	0.95
	Raman-brsvf (cm^{-1})	14.21	13.07	11.36	16.49	17.45	13.42	16.72	12.03	14.23	15.80	9.26	6.40	8.26	14.33	13.62	18.20	8.71	4.97	10.01	21.90	9.44	13.23	11.99	11.43	12.75	10.67	14.06	17.64	10.85
	Raman-msvf (cm^{-1})	10.95	11.09	8.03	7.23	6.15	9.26	7.76	9.88	8.80	7.92	2.87	8.65	4.69	10.64	8.48	3.68	9.51	5.77	6.35	29.70	9.98	10.16	9.08	5.46	6.90	6.45	4.00	8.20	19.06
Geometric	Q_{XX} (Debye·Å)	-167.73	-153.34	-127.03	-146.92	-159.64	-143.48	-150.15	-132.96	-175.20	-145.65	-122.88	-89.92	-128.10	-126.16	-151.07	-162.88	-160.77	-123.46	-172.64	-165.34	-184.94	-159.93	-135.52	-162.64	-158.35	-159.09	-140.19	-165.04	-162.64
	Q_{YY} (Debye·Å)	-184.31	-152.97	-134.89	-144.48	-147.31	-138.26	-163.15	-143.05	-162.54	-148.03	-127.23	-114.92	-135.34	-151.78	-161.41	-147.21	-148.07	-131.82	-152.17	-173.48	-152.92	-179.19	-136.59	-163.94	-155.14	-147.50	-153.05	-162.47	-163.94
	Q_{ZZ} (Debye·Å)	156.11	-148.29	-129.68	-140.28	-146.51	-137.38	-142.06	-132.24	-156.86	-148.51	-124.40	-105.06	127.71	-150.96	-151.35	-145.41	-148.01	-129.49	-145.09	-164.41	-158.59	-149.12	-133.46	-160.93	-144.46	-145.71	-142.14	-166.18	-160.93
	Q_{XY} (Debye·Å)	24.39	-28.72	-26.76	-23.13	26.51	24.37	23.91	27.19	24.46	-29.80	22.63	10.18	-26.53	-31.44	23.12	23.71	25.42	22.23	25.40	-31.63	31.92	-19.67	-32.17	25.18	-19.22	16.12	-26.80	8.99	25.18
	Q_{YZ} (Debye·Å)	3.02	-3.42	-3.02	-1.61	3.32	2.00	2.52	1.13	-0.77	-9.51	0.97	-0.27	3.10	-1.60	0.63	-0.20	4.83	0.01	-1.61	-5.28	4.15	-2.47	-1.45	0.03	2.11	2.55	-4.68	0.10	0.03
	MR (cm^3/mol)	105.36	99.23	86.98	92.08	97.28	92.02	92.36	89.39	105.73	95.48	83.92	66.23	85.24	96.93	101.33	97.28	98.07	81.99	97.52	108.43	104.00	100.32	87.73	102.69	98.09	99.98	99.58	100.41	105.42
	Mol Wt	399.40	359.40	319.30	351.30	361.40	333.40	369.30	331.30	389.40	362.40	303.30	262.20	320.30	357.40	375.40	361.40	363.40	318.30	360.40	401.40	392.40	385.40	352.30	393.80	379.80	359.40	395.40	409.80	417.40
Electronic	TE (aU)	-1401.27	-1227.01	-1110.31	-1248.85	-1262.93	-1149.61	-1348.06	-1148.38	-1341.51	-1278.93	-1043.19	-948.89	-1126.37	-1225.72	-1302.19	-1262.94	-1546.59	-1128.94	-1246.91	-1380.17	-1381.58	-1361.96	-1165.62	-1686.57	-1607.95	-1227.00	-1346.82	-1784.56	-1501.71
	q^+ (e)	0.53	0.53	0.53	0.53	0.53	0.53	0.53	0.53	0.53	0.53	0.70	0.58	0.53	0.53	0.53	0.53	0.53	0.53	0.53	0.53	0.52	0.53	0.53	0.53	0.53	0.52	0.53	0.54	0.61
	q^- (e)	-0.60	-0.60	-0.60	-0.60	-0.60	-0.60	-0.60	-0.60	-0.60	-0.60	-0.60	-0.58	-0.62	-0.60	-0.60	-0.60	-0.60	-0.73	-0.64	-0.60	-0.86	-0.60	-0.60	-0.72	-0.72	-0.60	-0.60	-0.72	-0.59
	E_{LUMO} (eV)	-0.05	-0.04	-0.04	-0.05	-0.05	-0.04	-0.05	-0.04	-0.05	-0.04	-0.06	-0.07	-0.05	-0.04	-0.04	-0.05	-0.05	-0.05	-0.05	-0.05	-0.05	-0.05	-0.05	-0.06	-0.06	-0.04	-0.05	-0.06	-0.05
	EG (eV)	0.16	0.17	0.17	0.17	0.16	0.16	0.16	0.17	0.16	0.16	0.16	0.15	0.16	0.16	0.16	0.16	0.15	0.17	0.17	0.17	0.15	0.16	0.16	0.16	0.16	0.16	0.17	0.16	0.15
Physical and Chemical	BP (K)	1033.78	963.83	928.83	951.29	972.88	911.33	914.85	958.45	1051.44	962.84	986.22	876.11	959.65	957.36	1026.94	972.88	975.55	966.07	1048.15	1079.44	1091.24	1051.28	942.87	1070.20	1043.05	1004.52	1003.37	1058.97	1073.74
	MP (K)	810.38	766.25	783.54	803.68	794.07	725.77	739.47	812.75	824.70	823.03	890.97	732.09	843.81	786.99	865.80	794.07	843.92	813.03	799.36	906.28	935.70	868.15	780.27	848.20	840.45	843.57	853.03	896.95	888.29
	CT (K)	992.57	956.77	942.87	939.48	960.37	930.53	919.97	960.23	982.63	965.76	966.22	909.95	949.60	953.62	984.24	960.37	978.82	932.21	973.63	1011.50	1009.04	1003.39	932.28	995.11	981.94	973.53	955.67	981.52	1002.65
	CV (cm^3/mol)	1010.50	945.50	831.50	904.50	910.50	876.50	912.50	844.50	1027.50	881.50	799.50	641.50	824.50	917.50	973.50	910.50	880.50	796.50	937.50	1042.50	1001.50	965.50	873.50	988.50	940.50	955.50	990.50	984.50	1038.50
	log*P*	2.98	2.04	1.37	1.84	1.35	1.74	1.75	1.32	1.74	0.51	0.50	0.71	1.46	1.52	1.51	1.35	1.60	0.53	1.79	1.68	1.31	2.60	-1.81	2.32	1.90	2.13	2.28	1.78	3.08
	HL	1.41	1.41	1.41	1.34	1.91	1.41	1.34	1.41	1.91	1.91	7.24	1.60	4.29	1.41	1.91	1.91	2.20	1.34	1.73	1.91	1.34	1.41	1.34	1.54	1.54	1.41	1.34	1.54	1.41
	HF (kJ/mol)	-500.76	-415.63	-462.39	-710.95	-538.46	-447.15	-850.84	-410.23	-620.52	-423.79	-147.95	-507.95	-408.96	-350.85	-615.54	-538.46	-320.22	-597.39	-673.36	-584.02	-677.45	-516.00	-580.62	-509.24	-482.44	-483.32	-907.35	-619.17	-764.56
	GE (kJ/mol)	-66.39	69.55	-51.63	-255.36	-34.62	-8.04	-407.29	17.54	-77.86	87.55	290.07	-159.98	17.00	146.79	-87.96	-34.62	90.65	-207.67	-207.49	-10.37	-128.66	-109.98	-131.51	-28.25	-24.57	17.04	-389.92	-139.21	-313.71

Table S2. The structures of 29 FQs.

No.	Compounds	Structure	No.	Compounds	Structure
1	Difloxacin	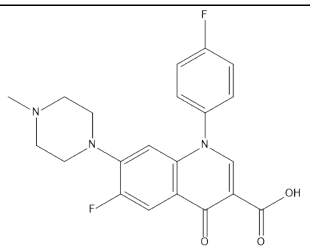	16	Levofloxacin	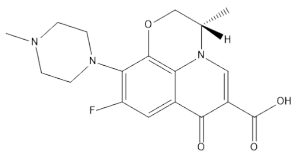
2	Enrofloxacin	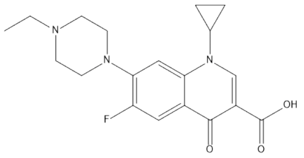	17	Rufloxacin	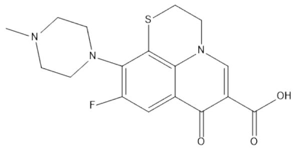
3	Norfloxacin	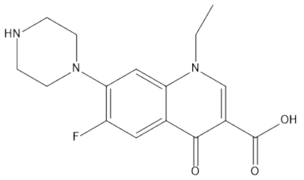	18	Pazufloxacin	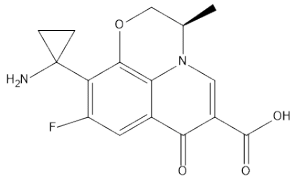
4	Lomefloxacin	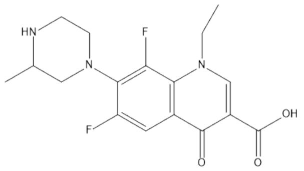	19	Nadifloxacin	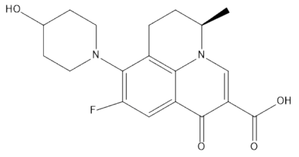
5	Ofloxacin	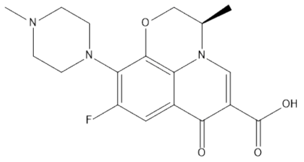	20	Moxifloxacin	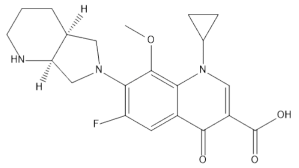
6	Pefloxacin	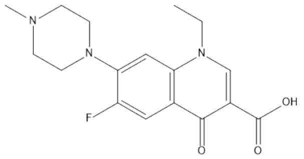	21	Sparfloxacin	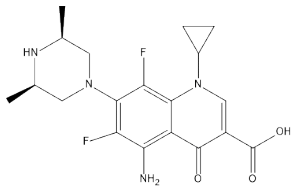
7	Fleroxacin	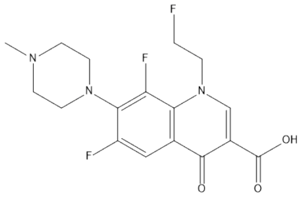	22	Sarafloxacin	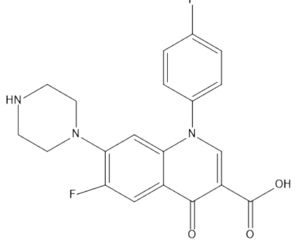
8	Ciprofloxacin	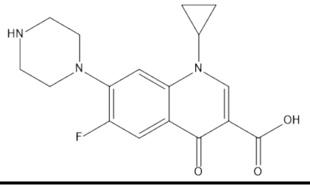	23	Amifloxacin	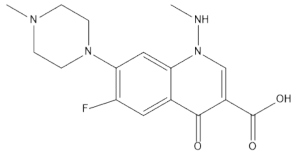

9	Balofloxacin	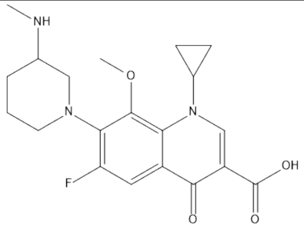	24	Besifloxacin	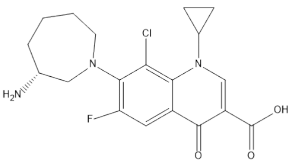
10	Marbofloxacin	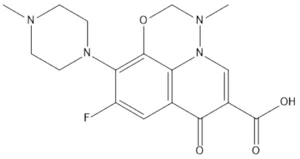	25	Clinafloxacin	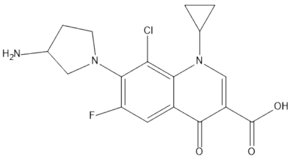
11	Pipemidic acid	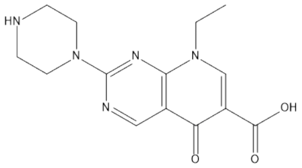	26	Grepafloxacin	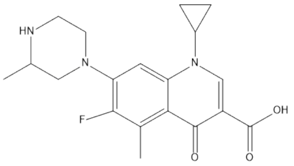
12	Cinoxacin	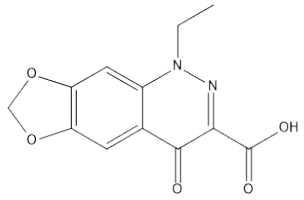	27	Orbifloxacin	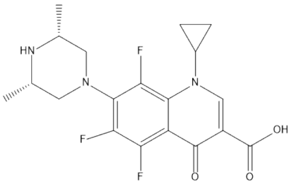
13	Enoxacin	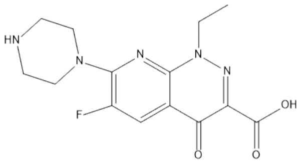	28	Sitafloxacin	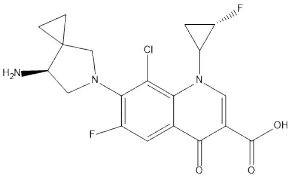
14	Danofloxacin	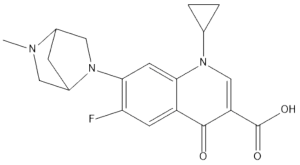	29	Temafloxacin	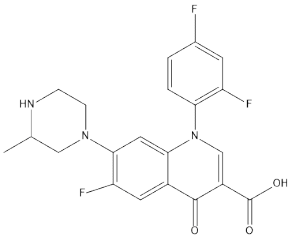
15	Gatifloxacin	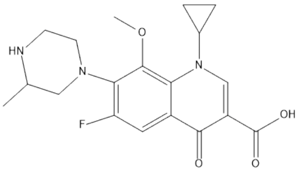			
